# Apparent prevalence and risk factors for bovine tuberculosis in the state of Paraná, Brazil: an assessment after 18 years since the beginning of the Brazilian program

**DOI:** 10.1007/s11250-022-03350-0

**Published:** 2022-10-24

**Authors:** Diego Leonardo Rodrigues, Elenice Aparecida Amorim, Fernando Ferreira, Marcos Amaku, Oswaldo Santos Baquero, José Henrique de Hildebrand e Grisi Filho, Ricardo Augusto Dias, Marcos Bryan Heinemann, Evelise Oliveira Telles, Vitor Salvador Picão Gonçalves, Christopher Compton, José Soares Ferreira Neto

**Affiliations:** 1 Ministry of Agriculture, Livestock and Supply of Brazil (MAPA), 420, José Veríssimo Street, Tarumã, Curitiba, Paraná Brazil; 2 Paraná Agribusiness Defense Agency (ADAPAR), 1559, Funcionários Street, Cabral, Curitiba, Paraná Brazil; 3 Department of Preventive Veterinary Medicine, University of São Paulo, 87, Prof. Orlando Marques de Paiva Avenue, São Paulo, Brazil; 4 EpiPlan, Faculty of Agronomy and Veterinary Medicine, University of Brasília, Ala Central Do Instituto Central de Ciências, Brasília, Distrito Federal Brazil; 5 The Epicentre, Massey University, Wool Building, University Avenue, Massey University Manawatu (Turitea), 4474 Palmerston North, New Zealand

**Keywords:** Tuberculosis, Prevalence, Risk factors, Paraná, Brazil, Cattle

## Abstract

Bovine tuberculosis (bTB) impacts considerably animal production and one health worldwide. To describe the prevalence, risk factors, and spatial pattern of the disease in the state of Paraná, Brazil, a cross-sectional study was conducted from September 2018 to February 2019. The area was divided into seven regions. Within each region, farms were randomly selected, and a predetermined number of cows was selected and tested by a comparative cervical tuberculin test. 17,210 animals were tested across 1757 farms. Herd prevalence of bTB-infected herds in Paraná was 2.5% [1.87–3.00%]. It has varied from 0.8 to 3.98% among seven regions, with clustering being detected in the west, central, and northeast areas. Animal prevalence was 0.35% [0.21–0.59%] and has varied from 0.08 to 0.6% among the pre-set regions. No major shifts in the prevalence of bTB were detected since 2007. Large-sized herds, dairy production, and feeding with whey were detected to be correlated with the presence of bTB. Exclusively among dairy herds, veterinary assistance from cooperatives, possession of self-owned equipment to cool milk, and feeding with whey were correlated with the disease. Considering these results, it is recommended that the state of Paraná seek to implement a surveillance system for the detection of bTB-infected herds transforming them into free ones, if possible, incorporating elements of risk-based surveillance. Health education is also recommended to inform farmers about the risks of introducing animals without testing and of feeding raw whey to calves.

## Introduction

Bovine tuberculosis (bTB) is a bacterial zoonosis caused by *Mycobacterium bovis* that is responsible for considerable losses in the dairy and beef sectors of cattle industries. Direct economic losses from bTB in animal production can be quantified in terms of the reduction of milk and meat production, condemned carcasses, and organs at the slaughterhouses and, eventually, cattle mortality. Cows found positive for the intradermal test were reported to produce 10% less milk on average, ranging from a reduction of 0.5 to 14.6% (Boland et al., 2010). Additionally, the World Health Organization states that at least 140,000 cases of human tuberculosis caused by *M. bovis* are reported annually around the world (WHO, 2021). Therefore, in addition to the animal health costs and animal welfare impacts, the effect of bTB on international trade and human health should be further considered to provide a more precise assessment of the burden of the disease (Azami and Zinsstag, 2018). These reasons have led many countries to implement measures to control or eradicate bTB.

Few countries have managed to eradicate the disease. Australia was considered free in 1997, and the path followed was described by More (2015). Netherlands, Sweden, Denmark, Finland, Estonia, Czech Republic, Latvia, Lithuania, Luxembourg, Slovakia, and Canada are considered officially free of bTB in cattle (OTF), with no cases registered in cattle for many years, while France, Germany, Austria, Poland, Belgium, Hungary, and Slovenia, are also OTF, but with few cases reported annually (EFSA—European Food Safety Authority, 2019). According to the World Organisation for Animal Health (WOAH), a country can declare itself free of bTB if it demonstrates through a surveillance system that in the last 3 years 99.8% of the premises were tested and found not infected (OIE, 2019).

On the other hand, many other countries have implemented strategies but with poor or unreported results. In some of them, economic and political constraints still play an important role and can limit the efforts to control bTB. In other cases, wildlife reservoirs have proved to be an obstacle to sustainable free status. Badgers in the UK, wild boar in parts of the EU, white-tailed deer in the USA, and brushtail possums in New Zealand are good examples of how those non-domestic bTB reservoir species are a barrier to control of bTB. Programs for control and eradication of bTB consist mainly of detecting infected herds and animals through surveillance systems, culling test-positive animals, and movement restrictions of infected herds. Some countries have additionally included control schemes for wildlife reservoir species.

The economic benefits of such programs should take into account the complexity of the direct and indirect impacts, both on animal and human health. A systematic review of the economic benefits of bTB control has shown many gaps of knowledge in this field but concluded that this classical approach using surveillance, culling, and movement restrictions is still viable and worth doing in the long-term scenario (Caminiti et al., 2016).

In Brazil, a national program for the control and eradication of bTB (PNCEBT) was established in 2001 (Brasil, 2001), and during its first years, herd and animal prevalence of the disease was obtained in many states of the country, ranging from 1.3 to 9.0% and from 0.03 to 1.3%, respectively (Alves et al., 2009; Barbieri et al., 2016; Silva et al., 2016; Veloso et al., 2016; Vendrame et al., 2016; Dias et al., 2016; Galvis et al., 2016; Guedes et al., 2016; Lima et al., 2016; Néspoli et al., 2016; Queiroz et al., 2016; Ribeiro et al., 2016; Rocha et al., 2016). In the state of Paraná, a study between 2005 and 2007 revealed an infected herd prevalence of 2.15% and an animal prevalence of 0.42% (Silva et al., 2016). Those studies indicated that the introduction of new animals to a herd without testing was a major risk factor for bTB in herds in Brazil (Ferreira Neto et al., 2016), while the presence of wild animal species on farms has not been associated with the disease so far.

Understanding the epidemiology of bTB is fundamental for the development of its control strategies. The efficiency of such strategies should be verified continually (Thrusfield and Christley, 2018). Resources expended and their respective results should be assessed to maximize the benefit–cost ratio of any measures adopted, and if necessary, alternative approaches should be explored. Considering all the measures used to control bTB in Brazil for 18 years between 2001 and 2018, it is necessary to evaluate their effectiveness. Thus, this study aimed to (i) assess the herd and animal prevalence of bTB in the state of Paraná, Brazil, after 18 years of the launch of PNCEBT, (ii) assess those parameters in seven areas of the state, (iii) compare results/prevalence with previous studies, and (iv) undertake an analysis of risk factors for bTB in cattle in this state.

## Materials and methods

This study was conducted in cooperation with the Brazilian Ministry of Agriculture, Livestock and Food Supply (MAPA), the Collaborating Centre on Animal Health of the Faculty of Veterinary Medicine and Animal Science of the University of São Paulo (FMVZ/USP), Epicentre—Massey University, New Zealand and the Animal Health Agency of the State of Paraná (ADAPAR). Fieldwork was carried out from September 2018 to February 2019 by ADAPAR.

### Study population

The target population was breeding female bovines and buffalos (for this text referred to simply as cattle or bovine) over 24 months of age. The state has a cattle population of 8,397,219 individuals, from which 4,126,775 are cows (i.e., target population) (IBGE, 2017). It represents 4.8% of the cattle in the country and has the third-largest dairy production by state.

### Sampling

**Fig. 1:**
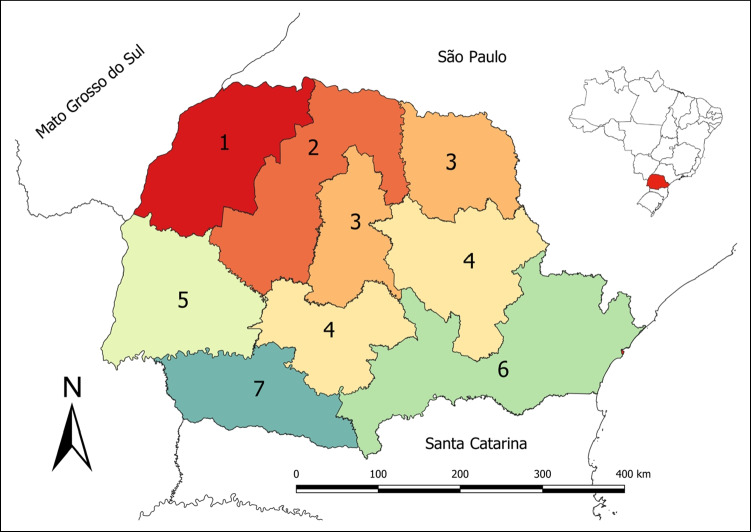
Map of the state of Paraná divided into seven regions: (1) Noroeste, (2) Centro-Oeste-Norte, (3) Norte pioneiro, (4) Centro-Sul, (5) Oeste, (6) Leste-Sul, and (7) Sudoeste, 2019

For sampling, the state was divided into seven administrative regions (Fig. 1) to characterize differences between production systems, management practices, breeds, average herd size, marketing methods, and sanitary practices. A cross-sectional study was performed in each region to investigate the prevalence of bTB-infected herds and animals by using a two-stage sampling method (Thrusfield and Christley, 2018). In the first stage, a predetermined number of farms with cows over 24 months were randomly selected (primary sampling units). In the second stage, within each selected farm, a predetermined number of female cattle aged over 2 years were selected (secondary sampling units).

The sample size of properties to be accessed in each region was estimated in accordance with the formula for simple random samples for large populations (Thrusfield, 2018):

$${\varvec{n}}=\frac{{{\varvec{Z}}}_{\boldsymbol{\alpha }}^{2}\boldsymbol{*}{\varvec{P}}\boldsymbol{*}\left(1-{\varvec{P}}\right)}{{{\varvec{d}}}^{2}}$$

*n* being the sample size, *Z* the normal distribution value at α level of confidence, *P* the expected prevalence, and *d* being the absolute error.

Sample size parameters were 0.95 confidence level, 0.2 of expected apparent prevalence, and 0.05 absolute error.

An exhaustive alphabetically sorted list of farms in each region was produced for the first stage of the sampling scheme. Then, systematic random sampling was applied. When the selected unit needed to be replaced, the next available propriety in the list was sampled.

In each randomly selected farm, a minimum number of cows over 24 months was tested to classify it as an infected or not infected herd. Freedom from disease formula was applied (Sergeant, 2018) to estimate if a farm was infected. The following parameters were used: the value of sensitivity and specificity of the test protocols (animal level) were 0.775 and 0.995, respectively (Lôbo, 2008), 15% of intra-herd prevalence, and an aggregate sensitivity and specificity greater than or equal to 0.90 (herd level). Considering those values, all cows were sampled in properties with up to 20 cows, and 20 animals were tested on farms with 21–99 cows, and 40 cows were sampled in properties with over 100 cows. The cut-off number of cows to define a property as infected for properties up to 99 cows was 1 infected animal (i.e., farms with 1 or more positives were considered infected) and for properties over 100 cows, the cut-off was 2 infected animals.

### Test protocol

A delayed hypersensitivity skin test using purified protein derivative (PPD) was used to test the selected cows. A comparative cervical skin test was undertaken using bovine and avian tuberculin simultaneously, with the test read after 72 h of inoculation, as described by WOAH and PNCEBT (Brasil, 2017; OIE, 2019).

### Calculation of apparent prevalence in herds and animals

The apparent prevalence of infected herds was calculated for each region as the proportion of positive herds divided by the number of tested herds. The apparent herd prevalence in the state of Paraná was based on the relative weight of each region as follows:

$$Herd\;weight =\frac{FR}{FSR}$$

where FR is the number of farms within the region, and FSR is the number of farms sampled in the region.

When calculating the animal prevalence in each region, we applied the following expression as the weight of each animal within that region:

$$Animal\;weight=\frac{CF}{CSF}$$

where CF is the number of cows in the farm, and CSF is the number of cows sampled in the same farm.

In order to calculate apparent animal prevalence in the Paraná, each sampled cow has its weight given by the following expression (Dohoo et al., 2003):

$$Animal\;weight\;for\;state\;level=\frac{CF}{CSF}*\frac{CR}{CSR}$$

where *CF* = number of cows on the farm; *CSF* = number of cows sampled on the same farm; *CR* = number of cows in the region; *CSR* = number of cows sampled in the region.

Herd prevalence in previous studies was compared with current values using a proportion test. Statistical analyses were performed using R (R Core Team, 2019). Confidence intervals (CI) were obtained by the binomial logistic regression model.

### Spatial analysis

A point pattern analysis to investigate the density variation of positive farms was performed by applying kernel density estimation applying the *density* function of the *stats* package of R (R Core Team, 2019), and bandwidth was selected according to the sampling density. Considering that sampling density was different among the 7 regions, a weighting process was applied to allow comparison across the whole state of Paraná. A sampling density in each of the seven regions was calculated and applied accordingly to undertake a pattern analysis across the whole state of Paraná (Wang et al., 2013). Next, a hypothesis test of significantly increased prevalence at a confidence level of 90% was conducted (Hazelton and Davies, 2009).

### Study of risk factors

Farm owners or farm managers answered a questionnaire. Questions included the number of bovines and buffalos by age and sex category, management and operation type, production practices, breeds, purchase, and sale of animals, use of calving pasture, indirect contact with other properties, presence of other domestic and wild species and veterinary care. All herds sampled provided one set of answers each. Data were collected in a face-to-face interview during the same visit to collect samples, using an electronic device, and then uploaded into an electronic spreadsheet.

Some variables with more than two possible answers were regrouped in some cases for a more meaningful analysis. The number of cows over 24 months in a herd was used as a measure of herd size, and the 3rd quartile was the cutoff point for categorization in two groups (small and large-sized herds).

All potential risk factors were submitted to univariate analysis regarding the farm bTB status applying the Chi-squared test or Fischer’s exact test. A conservative *p*-value of 0.2 or less was used as criteria to select which variables would be used in multivariate logistic regression; then, a backward process was used to eliminate the variables statistically not significant (Silva Abreu et al., 2009), and the Akaike information criterion (AIC) was applied to determine the best fitness. Considering the results obtained, we also applied the same methodology exclusively to the dairy farms in the sample. Risk factor analyses were performed using R (R Core Team, 2019).

## Results

Table 1 provides information regarding the target population and samples per region. Table 2 shows the apparent prevalence of bTB-infected herds in the state of Paraná.

**Table 1:** Population and sample data of the study per region in the state of Paraná, Brazil, 2019

Region	Nº of municipalities	Total herds with reproductive activity	Total cows	Herds sampled	Sampled cows
1. Noroeste	57	18,875	763,643	251	3406
2 Centro-Oeste-Norte	78	15,533	488,045	250	2673
3. Norte pioneiro	80	28,133	795,964	253	2352
4. Centro-Sul	32	26,011	729,383	251	2692
5. Oeste	52	23,153	583,813	251	2147
6. Leste-Sul	56	18,697	182,131	251	1450
7. Sudoeste	44	32,786	583,796	250	2490
Total	399	163,188	4,126,775	1757	17,210

**Table 2:** Apparent prevalence of bTB-infected herds in the state of Paraná, 2019

Region	Herds sampled	Herds positive	Prevalence (%)	CI (95%)
1. Noroeste	251	6	2.39	[0.49–4.28]
2. Centro-Oeste-Norte	250	9	3.6	[1.28–5.91]
3. Norte Pioneiro	253	6	2.37	[0.49–4.25]
4. Centro-Sul	251	10	3.98	[1.55–6.40]
5. Oeste	251	8	3.18	[1.01–5.36]
6. Leste-Sul	251	5	1.99	[0.25–3.72]
7. Sudoeste	250	2	0.8	[0–1.90]
Paraná State	1757	46	2.5	[1.87–3.0]

**Fig. 2:**
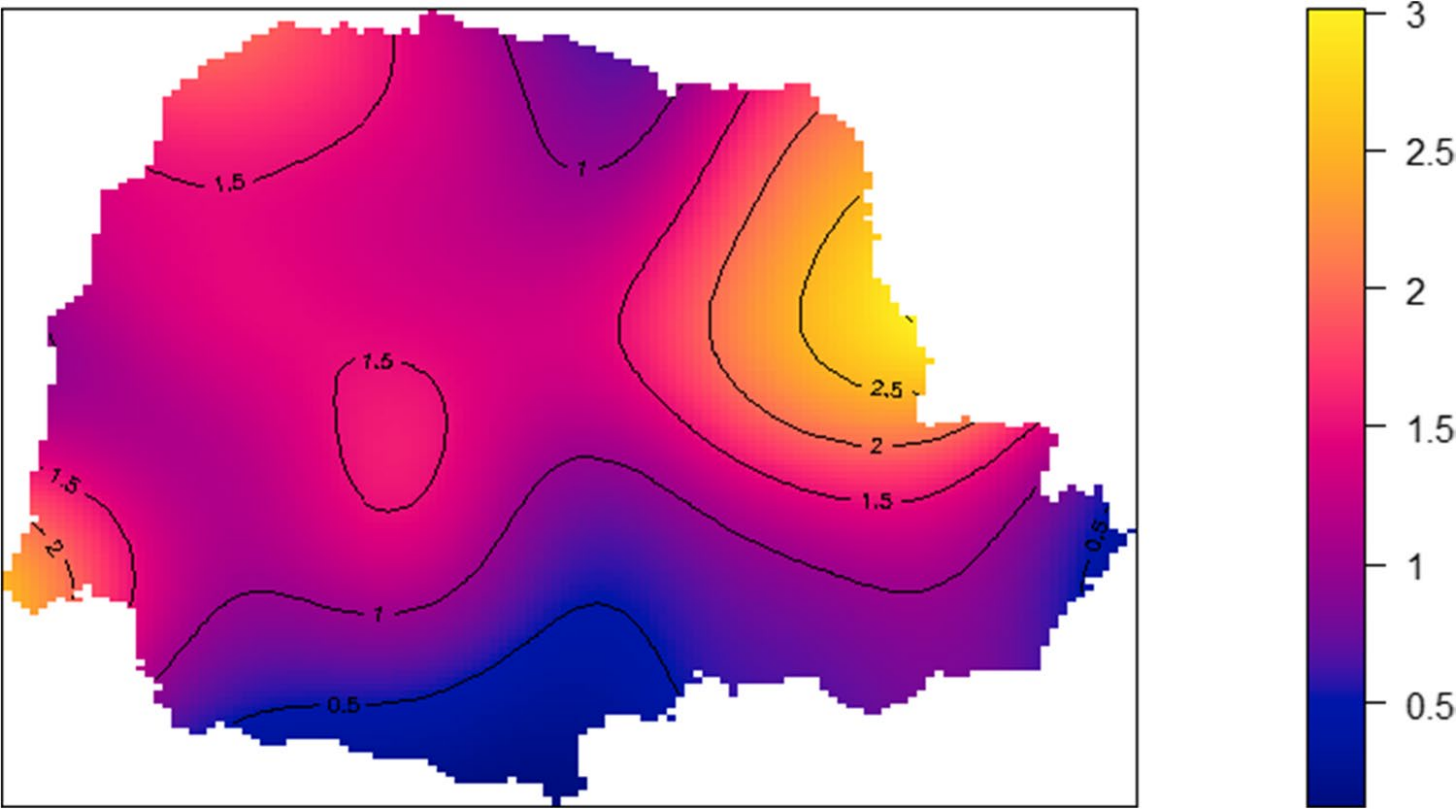
Spatial density of the bTB-infected farms across the state of Paraná, Brazil, 2019

**Fig. 3:**
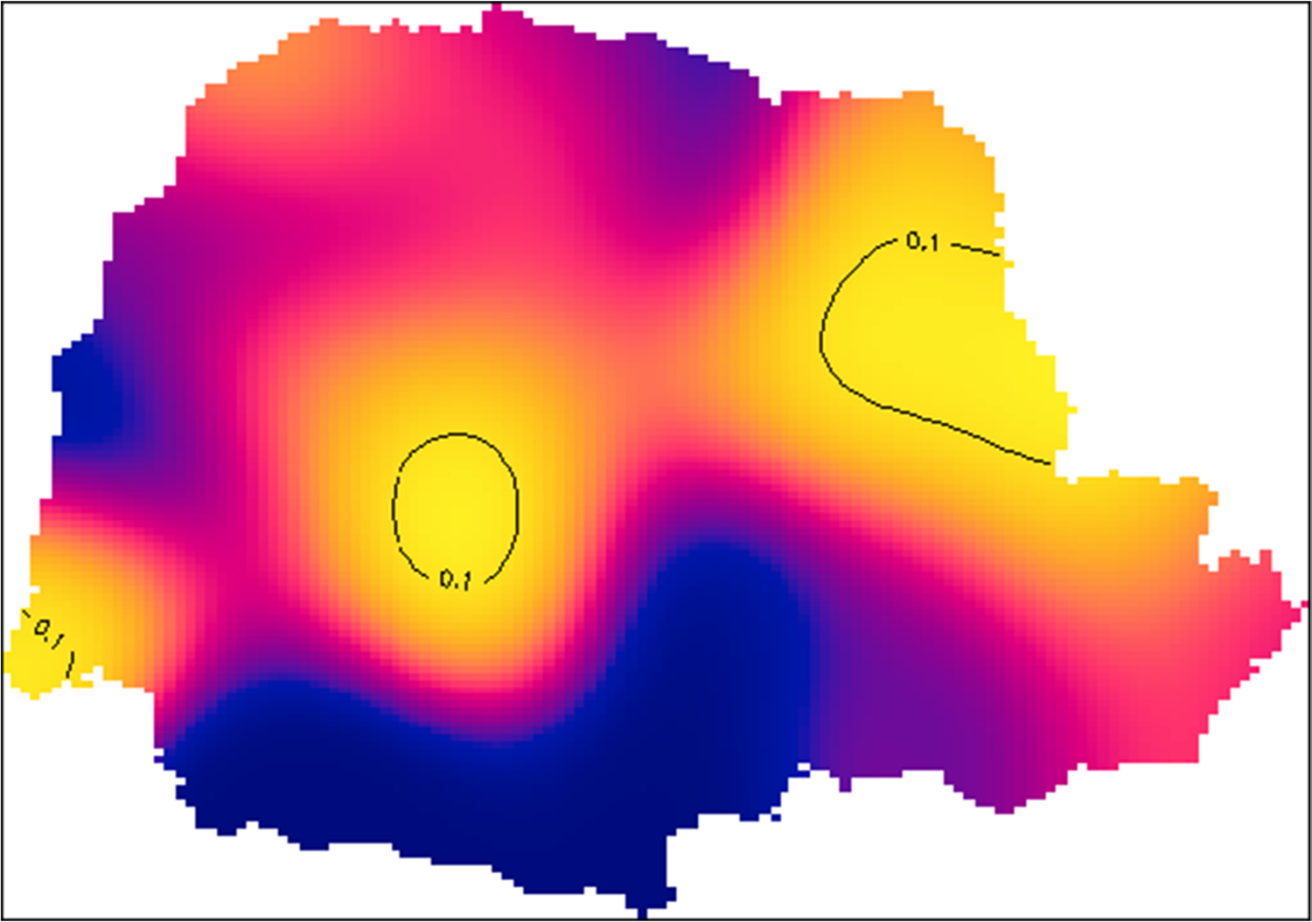
Areas with a significantly higher prevalence of bTB at a 90% confidence level across the state of Paraná, Brazil, 2019

The spatial density of infected farms in the state varied between 0.5 and 2.5 for the bandwidth selected and was higher in the east and western regions of Paraná (Fig. 2). Also, it was detected in some areas with a significantly higher prevalence at a 90% confidence level (Fig. 3).

Table 3 shows the apparent prevalence of bTB-infected herds in the regions, stratified by production type, and Table 4 reports the apparent prevalence of bTB in cows in the state of Paraná.

**Table 3:** Apparent prevalence of bTB-infected herds in the regions of the state of Paraná, stratified by type of production, 2019

Region	Beef		Dairy		Mixed	
	Prevalence%	CI (95%)	Prevalence%	CI (95%)	Prevalence%	CI(95%)
1	0.99 (1/101)	[0–2.92]	3.26 (3/92)	[0–6.89]	3.44 (2/58)	[0–8.15]
2	4 (3/75)	[0–8.44]	5.76 (6/104)	[1.27–10.25]	0 (0/71)	[0–4.08]*
3	0 (0/100)	[0–2.92]*	4.16 (4/96)	[0.16–8.17]	3.5 (2/57)	[0–8.29]
4	4.10 (3/73)	[0–8.67]	4.95 (5/101)	[0.7–9.18]	2.59 (2/77)	[0–6.15]
5	2.27 (1/44)	[0–6.68]	4.8 (6/125)	[1.04–8.55]	1.21 (1/82)	[0–3.59]
6	2.70 (2/74)	[0–6.40]	4.16 (3/72)	[0–8.79]	0 (0/105)	[0–2.79]*
7	0 (0/38)	[0–7.39]*	1.11 (2/179)	[0–2.66]	0 (0/33)	[0–8.43]*

*using beta distribution and Monte Carlo simulation.

**Table 4:** Apparent prevalence of bTB in cows in the state of Paraná, 2019

Region	Cows sampled	Cows positive	Prevalence (%)	CI (95%)
1. Noroeste	3406	15	0.2	[0–0.42]
2. Centro-Oeste-Norte	2673	14	0.4	[0.11–0.69]
3. Norte Pioneiro	2352	21	0.6	[0–1.42]
4. Centro-Sul	2692	13	0.37	[0.06–0.67]
5. Oeste	2147	9	0.41	[0.08–0.75]
6. Leste-Sul	1450	6	0.45	[0–0.96]
7. Sudoeste	2490	4	0.08	[0.04–0.21]
Paraná	17,210	82	0.35	[0.21–0.59]

**Fig. 4:**
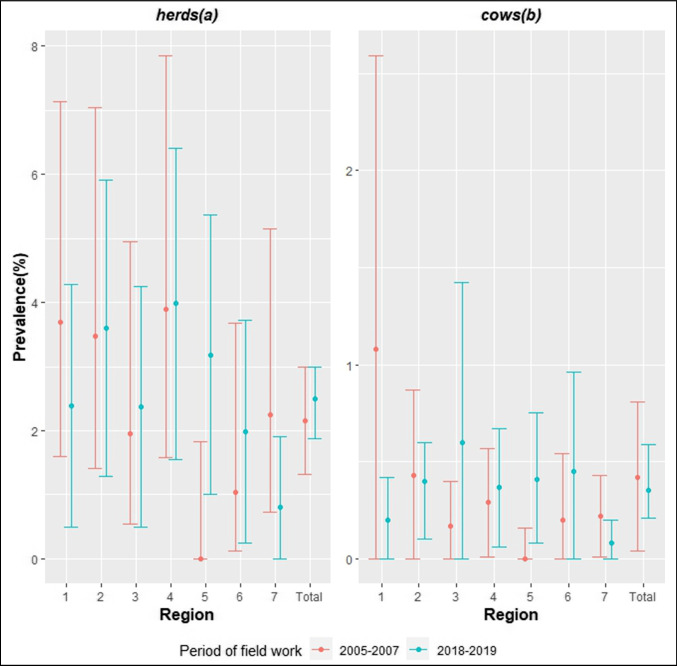
Apparent prevalence of bTB-infected herds (**a**) and cows (**b**) in the state of Paraná in the studies of 2005–2007 and 2018–2019 (present one)

Figure 4 compares the results of the present study with the previous one, whose fieldwork was conducted between 2005 and 2007 (Silva et al., 2016).

The variables selected by the univariate analysis (*p* ≤ 0.20) were the type of farm, herd size (number of cows over 24 m), presence of any wild species, bTB diagnosis, the introduction of the breeder in the last 2 years, slaughter bovines in the own farm, share only equipment with another farm, bovines concentrated in the shed, bovines concentrated in the salt trough, veterinary assistance, feeding with whey, and milking technique. Table 5 the final multivariate logistic regression model for the state.

**Table 5:** Results of final multivariate logistic regression model for bTB risk factors for farms from the state of Paraná, Brazil, 2019

Variable and levels	Odds ratio	95% CI	*p*-value
Herd size (number of cows over 24 m)			
≤ *19 (3*^*rd*^* quartile)*	Base category		
> *19*	2.11	1.14–3.91	0.01
Type of herd			
* Meat and mixed-type herd*	Base category		
* Dairy herd*	2.03	1.08–3.80	0.04
Use of whey to feed			
* No*	Base category		
* Yes*	5.31	1.85–15.29	0.00

Considering the risks identified above and that bTB is mainly found in dairy herds, we further explored risk factors for dairy farms only (*n* = 769; positive herds = 29). Thus, we established production characteristics that were more like (*p* < 0.2) to be seen in positive dairy farms: herd size (number of cows over 24 m), if their milk is sold to industry, veterinary assistance, feeding with whey, sort of milking, and if the milk cooling equipment is self-owned. Next, it was fitted with a logistic regression model that better suited our dataset (Table 6), following the same methodology described previously.

**Table 6:** Results of final multivariate logistic regression model for bTB risk factors for dairy farms from the state of Paraná, Brazil, 2019

Variable and levels	Odds ratio	95% CI	*p*-value
Veterinary Assistance			
No assistance or eventual private assistance	Base category		
Permanent veterinary assistance from Cooperatives	3.55	1.30–9.67	0.01
Milk cooling			
No cooling or community coolers	Base category		
Using self-owned equipment	3.06	1.07–8.75	0.03
Use of whey to feed			
No	Base category		
Yes	5.68	1.56–20.63	0.00

## Discussion

The apparent prevalence of infected herds among regions has ranged from 0.8 (region 7) to 3.98 (region 4) (Table 2), and there was a significant difference between regions 7 and 4 (*p* = 0.02) and 7 and 2 (*p* = 0.03). There is a tendency for lower values in regions 6 and 7, bordering the state of Santa Catarina (Table 2).

The overall apparent prevalence of bTB among herds in Paraná was 2.5% [1.87; 3.0] (Table 2), equal to that in the states of Bahia (Bahiense et al., 2016), the Rio Grande do Sul (Queiroz et al., 2016), Mato Grosso do Sul (Guedes et al., 2016), Mato Grosso (Néspoli et al., 2016), Rondônia (Vendrame et al., 2016), Goiás (Rocha et al., 2016), Pernambuco (Lima et al., 2016), and Distrito Federal (Ribeiro et al., 2016), higher than that in the states of Santa Catarina (Veloso et al., 2016) and Tocantins (Ferreira Neto et al., 2021) and lower than that reported for São Paulo (Dias et al., 2016), Minas Gerais (Barbieri et al., 2016), and Espírito Santo (Galvis et al., 2016).

No significant changes occurred in the herd-level prevalence of bTB in the state of Paraná between 2007 (Silva et al., 2016) and 2019 (present study). However, when looking at each of the seven regions individually, a significant increase in positive herd prevalence from 0 to 3.18% was detected in region 5 (*p* = 0.02). Considering the stability in the prevalence of bTB seen in the last 12 years, it is recommended that strategies adopted to control it should be assessed and reviewed.

It is useful to view those figures geographically and compare them with other states bordering with Paraná. Thus, in the southern state of Santa Catarina, herd prevalence ranges from 0 to 1.30% (Veloso et al., 2016), close to those found in the southern areas of Parana: 0.8% (region 7) and 1.99% (regions 6) (Table 2). Conversely, in the Northeast areas (regions 2, 3, and 4) higher prevalence was detected (3.6%, 2.3%, and 3.9%, respectively), and all of them bordering the state of São Paulo which has the highest herd prevalence in Brazil—9% (Dias et al., 2016). Thereby, the influence of adjacent administrative areas should be taken into account when defining strategies of control, considering especially the network of cattle trade in the area (Amaku et al., 2015).

Spatial analysis of the data suggests heterogeneity across the region, with the extreme West, Center, and Northeast areas of Paraná showing a higher density of bTB-positive farms area (Figs. 2 and 3). Those clustering areas tend to be an important source of bTB to other areas of the state, and their cattle trade should be considered a higher risk.

Larger-sized farms, dairy-type herds, and feeding whey to cattle were identified as risk factors for infection with bTB (Table 5). Herd size has been consistently identified as an important risk factor using different methodologies in different countries (Dias et al., 2016; Galvis et al., 2016; Néspoli et al., 2016). Production practices in larger-sized herds differ from the smaller ones and may explain this relationship. They are more likely to replace animals with purchased stock which increases the risk to introduce *M. bovis* (Gilbert et al., 2005)*.* According to our sample, from 822 smaller herds, only 298 (36.25%) reported the introduction of bovines, while in the 935 larger herds, 458 (48.98%) did. This is in line with outbreaks investigations in Northern Ireland, which reported that 15–30% of them were caused by the purchase of infected animals (Menzies and Neil, 2000). In bTB, this risk factor is key to understanding the dynamics of the disease, considering that once infected, animals can potentially be a source of infection for their remaining life (Conlan and Wood, 2018). Producers may reduce that risk by carefully testing any bovines before introduction onto their properties.

The type of cattle farming enterprise is also a risk factor for herd bTB infection status. Cows in dairy farms are frequently reared in confined areas compared with beef farms which are more commonly managed outdoors. Milking parlors and sheds are areas within farms with high rates of contact between cows and direct transmission of *M. bovis* between cows occurs (Chopra et al., 2020). The main route of transmission of bTB is via the respiratory system (Cassidy, 2006), and therefore, infected cows kept in high-density groups are more likely to infect other cows than those that are reared in extensive grass-fed beef systems. Accordingly, 3.8% (29/769) of dairy herds were bTB positive against only 1.7% (17/971) of beef or mixed ones. The data in Table 3 corroborate the importance of dairy farms in bTB transmission. In Brazil, dairy herds also presented an increased risk for bTB in the state of Rio Grande do Sul, Mato Grosso São Paulo, and Santa Catarina (Dias et al., 2016; Néspoli et al., 2016; Queiroz et al., 2016; Veloso et al., 2016), and the same was verified in New Zealand (Porphyre et al., 2008).

A third factor found to be relevant was the use of whey to feed cattle. Milk derivates thermically unprocessed may be a route of infection for *M. bovis* (Conlan and Wood, 2018). It would be useful to investigate the source of this feed component. It is expected that the oral route of infection will produce lesions in the abdominal cavity (Neill et al., 1994), and while mesenteric lymph node bTB lesion is considered rare in developed countries, it still plays a considerable role in Brazil (Grisi-Filho et al., 2011; Ramos et al., 2018).

To better inform a risk-based surveillance program for the state, the risk factors for bTB infection were analyzed among dairy farms only. Previous evidence is that dairy farms that are more technically developed pose a greater risk to bTB (Ferreira Neto et al., 2016). Similarly, our model (Table 6) has shown that having their own cooling equipment at the farm, more intense assistance from veterinary services, and the use of whey are characteristics associated with positive status for bTB. More intensively managed dairy farms are drivers for bTB as they tend to reduce the space available per animal on the farm and, as discussed previously, allow greater contact among animals (Chopra et al., 2020).

About veterinary care, it is important to highlight that our results show that properties belonging to cooperatives had a greater chance of being infected with tuberculosis, indicating that the disease is more concentrated in dairy cooperatives. Those production sets have different practices when compared with smaller producers, including a more intense trading pattern, which may explain this difference.

Given the limitation of cross-sectional studies to detect risk factors for diseases (Dohoo et al., 2003), especially in low prevalence scenarios, it is recommended to apply classic case–control studies to better individualize the risk factors for bTB in Paraná, aiming to provide greater certainty for the incorporation of risk-based surveillance elements into future strategies to fight the disease.

Considering these results, it is recommended that the state of Paraná seek to implement a surveillance system for the detection of bTB-infected herds, transforming them into free ones, if possible, incorporating elements of risk-based surveillance. Health education is also recommended to inform farmers about the risks of introducing animals without testing and of feeding raw whey to calves.

## Data Availability

The datasets generated during and/or analyzed during the current study are not publicly available due to personal content but may be made available from the corresponding author at reasonable request.
